# Supplementary material for: Transcranial direct current simulation as an adjunctive treatment for treatment-resistant depression in hospitalized patients: A feasibility study protocol
Source: PLoS One. 2025 Jun 10;20(6):e0324808. doi: 10.1371/journal.pone.0324808 (PMC12151435; doi:10.1371/journal.pone.0324808)
Supplement: S2 File — Full study protocol. (PDF) [file pone.0324808.s002.pdf]

A Feasibility Study of Transcranial Direct Current Stimulation (tDCS) as an Adjunctive Treatment for Treatment Resistant Depression (TRD) in Hospitalized Patients

**Principal Investigator:**	Maria I. Lapid, M.D., Professor of Psychiatry
**Co-PI:**	John W. Li, M.D., Ph.D., PGY-2 Psychiatry Resident
**Co-Investigators:**	Amanda M. Anderson, M.S.N., R.N., Instructor in Nursing<br>Michael R. Basso, Ph.D., L.P., Professor of Psychology<br>Paul E. Croarkin, D.O., M.S., Professor of Psychiatry and Pharmacology<br>Carly B. Erickson, R.N., Department of Nursing, Generose 3W<br>Misty N. Hemm, M.S.N., R.N., RN-BC, NS, Department of Nursing, Generose 3W<br>Ashley L. Holland, D.O., Instructor in Psychiatry, MCHS Eau Claire<br>Emily A. Jazdzewski, M.A.N., R.N., Instructor in Nursing, Nurse Manager, Generose 3E<br>Eduardo Kabristante, M.S.N., R.N., CRRN, Department of Nursing, Generose 3E<br>Simon Kung, M.D., Associate Professor of Psychiatry<br>Paul H. Min, Ph.D., Associate Professor of Radiology, Assistant Professor of Biomedical Engineering and Neurosurgery<br>Justine M. Parsons, M.B.A., R.N., Instructor in Nursing, Nurse Manager, Generose 3W<br>Caili Ren, M.D., Visiting Research Fellow<br>Roberta L. Sheldon, M.S., R.N., Instructor in Nursing<br>Erik K. St. Louis, M.D., Professor of Medicine and Neurology<br>Kirk M. Welker, M.D., Associate Professor of Radiology<br>Gregory A. Worrell, M.D., Ph.D., Professor of Neurology
**Biostatistician:**	Vanessa M. Pazdernik, M.S.
**Protocol Development Specialist:**	Barbara A. Hall
**Clinical Study Coordinators:**	Can Ozger<br>Daniel G. Smith<br>Sarah M. Williams
**Funding Sponsor:**	Department of Psychiatry & Psychology Small Grants Program 2023
**External Collaborator:**	Yogatheesan Varatharajah, Ph.D., Neurology Research Fellow

**Study Product:**	Soterix Medical 1×1 Low Intensity Transcranial DC Stimulator
**Protocol Number: (IRBe)**	23-003274
**IDE Number:**	N/A

Initial version: 09/11/23 Version **(1.0)** *[Update version number and version date in header also]*
Revised: 3/2/2024 Version **(2.0)**
Revised: [date] Version **(3.0)**
(Continue to add any additional amendment dates as protocol is modified and change version date as necessary)

Table of Contents

LIST OF ABBREVIATIONS 5
STUDY SUMMARY 6
1 INTRODUCTION 7
1.1 BACKGROUND 7
1.2 INVESTIGATIONAL DEVICE 8
1.3 CLINICAL DATA TO DATE 8
1.4 STUDY RATIONALE AND RISK ANALYSIS (RISKS TO BENEFITS RATIO) 9
1.4.1 Study Rationale 9
1.4.2 Anticipated Risks 10
1.4.3 Potential Benefits 12
1.5 ANTICIPATED DURATION OF THE CLINICAL INVESTIGATION 12
2 STUDY OBJECTIVES 12
2.1 PRIMARY OBJECTIVE 12
2.2 EXPLORATORY OBJECTIVE 12
3 STUDY DESIGN 13
3.1 GENERAL DESIGN 13
3.2 PRIMARY STUDY ENDPOINTS 14
3.3 EXPLORATORY STUDY ENDPOINTS 14
3.4 PRIMARY SAFETY ENDPOINTS 15
4 SUBJECT SELECTION, ENROLLMENT AND WITHDRAWAL 15
4.1 INCLUSION CRITERIA 15
4.2 EXCLUSION CRITERIA 15
4.3 SUBJECT RECRUITMENT, ENROLLMENT AND SCREENING 16
4.4 EARLY WITHDRAWAL OF SUBJECTS 16
4.4.1 When and How to Withdraw Subjects 16
4.4.2 Data Collection and Follow-up for Withdrawn Subjects 16
5 STUDY DEVICE 17
5.1 DESCRIPTION 17
5.2 METHOD FOR ASSIGNING SUBJECTS TO TREATMENT GROUPS 18
5.3 PREPARATION AND ADMINISTRATION/IMPLANTATION OF INVESTIGATIONAL DEVICE 19
5.4 SUBJECT COMPLIANCE MONITORING 22
5.5 PRIOR AND CONCOMITANT THERAPY 22
5.6 PACKAGING AND LABELING 23
5.7 MASKING/BLINDING OF STUDY 23
5.8 RECEIVING, STORAGE, DISTRIBUTION AND RETURN 23
5.8.1 Receipt of Investigational Devices 23
5.8.2 Storage 24
5.8.3 Distribution of Study Device 24
5.8.4 Return or Destruction of Study Device 24
6 STUDY PROCEDURES 24
VISIT 0: SCREENING VISIT 25
VISIT 1 - BASELINE ASSESSMENT VISIT* 25
VISIT 2-10 – TREATMENT VISITS 26
VISIT 10 – POST-TREATMENT VISIT 26
VISIT 11 – 1-MONTH FOLLOW UP VISIT 27
VISIT 12 – 6-MONTH FOLLOW UP VISIT ..... ERROR! BOOKMARK NOT DEFINED.
SCHEDULE OF EVENTS AND TIMELINE 27

7 STATISTICAL PLAN ... 28
7.1 SAMPLE SIZE DETERMINATION ... 28
7.2 STATISTICAL METHODS ... 28
7.3 SUBJECT POPULATION(S) FOR ANALYSIS ... 29
8 SAFETY AND ADVERSE EVENTS ... 30
8.1 DEFINITIONS ... 30
8.2 RECORDING OF ADVERSE EVENTS ... 32
8.3 SPONSOR-INVESTIGATOR REPORTING OF UNANTICIPATED ADVERSE DEVICE EFFECTS AND UNANTICIPATED PROBLEMS ... 33
8.3.1 Sponsor-Investigator Reporting, Notifying Mayo IRB ... 33
8.4 UNBLINDING PROCEDURES (BREAKING THE BLIND) (AS NECESSARY IF THE STUDY IS BLINDED) ... 34
8.5 MEDICAL MONITORING ... 34
9 DATA HANDLING AND RECORD KEEPING ... 34
9.1 CONFIDENTIALITY ... 34
9.2 SOURCE DOCUMENTS ... 34
9.3 CASE REPORT FORMS ... 34
9.4 RECORDS RETENTION ... 35
10 STUDY MONITORING, AUDITING, AND INSPECTING ... 35
10.1 STUDY MONITORING PLAN ... 35
11 ETHICAL CONSIDERATIONS ... 37
12 STUDY FINANCES ... 37
12.1 FUNDING SOURCE ... 37
12.2 CONFLICT OF INTEREST ... 37
12.3 SUBJECT STIPENDS OR PAYMENTS ... 38
13 PUBLICATION PLAN ... ERROR! BOOKMARK NOT DEFINED.
14 REFERENCES ... 38

List of Abbreviations

AE	Adverse Event/Adverse Experience
CFR	Code of Federal Regulations
CRF	Case Report Form
DSMB	Data and Safety Monitoring Board
FDA	Food and Drug Administration
fNIRS	Functional near-infrared spectroscopy
HIPAA	Health Insurance Portability and Accountability Act
IDE	Investigational Device Exemption
IRB	Institutional Review Board
PHI	Protected Health Information
PI	Principal Investigator
SAE	Serious Adverse Event/Serious Adverse Experience
tDCS	Transcranial Direct Current Stimulation
TRD	Treatment Resistant Depression
UADE	Unanticipated Adverse Device Effect

Study Summary

Title	A Feasibility Study of Transcranial Direct Current Stimulation (tDCS) as an Adjunctive Treatment for Treatment Resistant Depression (TRD) in Hospitalized Patients
Running Title	tDCS in TRD
IRB Protocol Number	23-003274
Phase	Pilot
Methodology	Open-label treatment
Overall Study Duration	1-2 weeks
Subject Participation Duration	1-2 weeks (baseline assessments/treatment/post-treatment assessments)
Objectives	The proposed study seeks to assess the feasibility, acceptability and tolerability of tDCS in adults with treatment resistant depression in the psychiatric hospital. We will assess the preliminary effects of tDCS on depressive and cognitive measures. We will also explore electroencephalographic and cerebral hemodynamic changes with tDCS treatments.
Number of Subjects	10
Diagnosis and Main Inclusion Criteria	1. Age 18 years and older<br>2. Clinical diagnosis of treatment-resistant depression<br>3. Voluntary admission status<br>4. Moderate or severe depression, defined by PHQ-9 ≥ 15
Study Device	Soterix Medical 1×1 Low Intensity Transcranial DC Stimulator Model 1300A<br>Stimulates cerebral cortex at low intensity (up to 2 mA) resulting in changes in cortical excitability and neural plasticity
Duration of Exposure	10 treatment sessions (2 mA, 30 minutes per session, twice daily) over 5 days
Reference therapy	Not applicable
Statistical Methodology	Within-subjects, repeated-measures analysis

1 Introduction

This document is a protocol for a human research study. This study will be carried out in accordance with the procedures described in this protocol, applicable United States government regulations and Mayo Clinic policies and procedures.

1.1 Background

Treatment Resistant Depression

Depression is a public health problem and one of the leading causes of disability worldwide. According to the National Institute of Mental Health, in 2020 an estimated 21 million adults in the United States had at least 1 episode of major depression in the past year, which represents 8.4% of the adult population. [1] Across the world, according to the World Health Organization, there are approximately 280 million people who have depression, and about 5% of the worldwide adult population have depression. [2] While current pharmacologic and nonpharmacologic treatments for depression are available, there is a significant subset of patients who do not respond to the usual treatments. Treatment-resistant depression (TRD) refers to this condition where an individual experiences little or no symptom relief despite 2 or more treatment attempts that are considered adequate trials. The prevalence of TRD varies greatly, given wide variations in definition, study populations, and inter-and intra-individual differences. Among adults treated for depression, the prevalence of TRD ranged from 6-55% across studies. [3, 4] There is a profound negative impact of TRD on individuals, families, and societies, with suicide being the most severe negative impact.

Noninvasive Brain Stimulation for Depression

In the context of continued search for effective treatments for TRD, novel nonpharmacologic approaches such as noninvasive brain stimulation (NIBS) have been increasingly utilized as primary or adjunctive treatments. NIBS involves stimulating specific areas of the brain to induce neuronal changes and eventually modulate neural circuits, which are thought to regulate mood or other neuropsychiatric symptoms. The two most common forms of NIBS are transcranial magnetic stimulation (TMS) which involves use of electromagnetic fields and transcranial direct current stimulation (tDCS) which involves use of electric currents. [5] While TMS is already FDA-approved for treatment resistant depression, tDCS is still investigational as a treatment for major depressive disorder and other neuropsychiatric conditions. In order to stimulate the brain transcranially with tDCS, a low-intensity electric current is delivered directly to the scalp through electrodes positioned on the head held in place by a headband, and the electric current is generated from a portable battery-operated device. In contrast to TMS where electromagnetic pulses lead to action potentials, the weak current used in tDCS does not stimulate action potentials but instead changes the voltage of neuronal resting membrane potential toward depolarization after anodal stimulation (excitatory) and toward hyperpolarization after cathodal stimulation (inhibitory), which alters the likelihood of action potentials firing. [6]

Efficacy of tDCS on Depression

In a systematic review of tDCS for the treatment of depression, treatment parameters used across studies included ranges of 1-2 mA current intensity, 25-35 cm2 electrode size, F3 (anodal) electrode position, right supraorbital (cathodal) reference electrode position, 20-30 minutes once or twice per day treatments, with a total of 5-15 stimulations over 1-3 weeks treatment duration. [7] Results of tDCS studies show inconsistent efficacy due to small sample sizes and heterogeneity of study populations. A recent meta-analysis showed modest effect of tDCS compared to sham ((k=25, Hedges'sg= 0.46, 95% confidence interval [CI]: 0.22–0.70) in treating depressive episodes. [8]

Peak Alpha Frequency as a Marker of Response to tDCS

In the study of TRD, peak alpha frequency (PAF) has received attention as a potential neurophysiological marker that provides insight into individual brain activity patterns that may influence responsiveness to noninvasive brain stimulation therapies such as tDCS. PAF represents the dominant frequency within the alpha band (typically between 8-12 Hz) where the alpha rhythms achieve maximum power. [9] This is measured via electroencephalogram (EEG) and has been implicated in a variety of cognitive and affective processes. [10] In the context of TRD, there is a growing interest in the potential of PAF as a predictor or correlate of treatment response. Given that tDCS exerts its effects through modulating cortical excitability and neural plasticity, exploring correlations between PAF and tDCS responsiveness in TRD patients could offer valuable insights. [11-13] Capturing PAF data via EEG, therefore, may help elucidate the neurophysiological mechanisms underlying the therapeutic impact of tDCS.

1.2 Investigational Device

The Soterix Medical 1×1 Low Intensity Transcranial DC Stimulator Model 1300A is a device that delivers low-intensity electrical current to the scalp with the aim of modulating brain function noninvasively. Stimulation is applied via two electrodes (an anode and a cathode) covered by saline-soaked sponges that are held against the scalp by a pair of large, adjustable elastic bands or head straps. During stimulation, a small current (2 mA or less) is applied to the scalp, resulting in a smaller amount of current reaching the underlying cerebral cortex. The device is powered by two 9-volt alkaline batteries. Please see additional details about the investigational device in Section 5.

1.3 Clinical Data to Date

Review of a total of 13 randomized controlled trial and open-label studies, which comprised of 947subjects with a mean age 47 years (range 18-75) showed that there is favorable evidence suggesting that tDCS is clinically effective in treating TRD as measured by response, symptom improvement, and disease remission in comparison to sham treatment. Additionally, tDCS has shown to enhance cognition and has the potential to augment psychotherapy. Further studies are needed to determine treatment benefit in currently psychiatrically hospitalized TRD patients.

The most common method of stimulation is anode placed over F3 and cathode placed over F4 in accordance with the International 10-20 Electroencephalogram System standard. Active tDCS sessions comprised of ramp-up phase (15-30 seconds) followed by 30 minutes of 2 mA intensity and ramp-down phase (15-30 seconds). Earlier studies also employed shorter duration of 20 minutes and lower current of 1 mA. Treatment frequency varies from twice daily to 3 times per week and duration varies from 5 days to 20 days. Two of the 13 trials (15%) reported tDCS to be not efficacious. Both were early trials with a relatively small sample size (n = 24 and 22).[14, 15] Later studies with similar sample size showed favorable results with tDCS intervention. More recent studies with a larger sample size in the hundreds showed non-inferiority compared to either selective serotonin reuptake inhibitor or cognitive behavior therapy. [16-18] The most common site of stimulation is using the anode over the left dorsal lateral prefrontal cortex. [19] The main adverse effects of this method seem to be redness of the skin, itching, headache, paresthesia (burning and tingling) at the stimulation site. Incidence of the side effects does not seem to be associated with repeated sessions of tDCS.[20]

1.4 Study Rationale and Risk Analysis (Risks to Benefits Ratio)

1.4.1 Study Rationale

Some tDCS studies included TRD populations, but no studies have been conducted in inpatient settings. Patients hospitalized psychiatrically for TRD have high severity of symptoms, more failed prior treatments, and are at high risk for suicide and disability. Most of these patients feel they have tried enough medications and want to try a non-medication treatment approach. Hospitalization provides a window of opportunity to test whether adding tDCS as an adjunctive treatment to standard inpatient psychiatric care for TRD could be a viable option. In addition to offering patients a novel treatment and more hope for getting better, it could potentially decrease hospital length of stay.

We therefore propose to test the feasibility, acceptability and tolerability of using tDCS in the inpatient setting with TRD patients, explore the utility of EEG biomarkers to assess response to tDCS, and assess preliminary impact on depressive and cognitive symptoms. This proposed pilot project will leverage existing equipment and resources within the Depression Center, which includes a Soterix tDCS platform and a CGX wireless EEG system that will be used to generate a peak alpha frequency (PAF) as a potential biomarker to assess response to tDCS.

For our study purposes, we define TRD as "depression that does not remit following two or more treatment attempts of an adequate dose and duration of a minimum duration of 4 weeks." This is the evidence-based definiton from the publication "Definition of Treatment-Resistant Depression in the Medicare Population" produced by the Agency for Healthcare Research and Quality (AHRQ) through its Evidence-based Practice Centers (EPCs) in response to the request of Centers for Medicare & Medicaid Services (CMS). [21]

1.4.2 Anticipated Risks

The adverse effects (AEs) of tDCS have been described in several systematic reviews of adult tDCS studies. Brunoni et al. [11] reviewed 209 tDCS studies, the majority of which involved healthy control subjects and single-session tDCS; of these, 74 studies (with 1851 subjects) included reporting of AEs. The authors noted that tDCS appeared well-tolerated overall, with relatively mild AEs (scalp itching in 39.3%; tingling sensations, 22.2%; headache, 14.8%; scalp discomfort, 10.4%; burning sensations, 8.7%) in those receiving active tDCS, and with no difference in AE rates between those receiving active and sham treatments. Aparício et al. [22] presented an updated systematic review of 64 randomized controlled trials (2262 subjects) whose protocols involved five or more treatment sessions. By contrast, the majority of studies included in this review consisted of samples with neurologic and psychiatric conditions. Subjects receiving active tDCS did not drop out of studies at higher rates than those receiving sham, and nearly half of studies reported no dropouts, indicating good acceptability of tDCS over multiple sessions. Another recent review by Bikson and colleagues [23] noted that over 33,200 sessions of tDCS have been performed in human subjects across a range of treatment parameters, without any serious adverse events (SAEs) or evidence of irreversible injury reported.

One systematic review of AEs in pediatric tDCS studies has been published. Krishnan and colleagues [24] reviewed 16 studies that utilized transcranial current stimulation (both tDCS and transcranial alternating current stimulation); all but one included reporting of AEs. The AEs reported, in order of decreasing frequency, included: tingling sensations (11.5%), itching (5.8%), skin erythema (4.7%), scalp discomfort (3.1%), mood changes (3.1%), fatigue (2.1%), headache (1.0%), burning sensations (1.0%), and sleepiness (1.0%). The authors noted that AEs related to discomfort at the electrode sites were transient and resolved within two hours of stimulation.

Risks of tDCS

The tDCS treatment is non-invasive and serious adverse events are rare. However, subjects may experience adverse effects of tDCS. The main side effects could be redness of the skin or skin lesions, itching or scalp itching, scalp discomfort, tingling or burning sensations, sensation of a short light flash, dizziness, headache or pain, mood or sleep changes, nausea or fatigue. Mild redness or lesions of the skin can occur under the electrodes and typically fades shortly after the session. Itching, discomfort, tingling or burning sensations can occur at the site of electrode placement. If these occur, we will reduce the current intensity, reposition the electrodes to the subjects' comfort, and ensure the electrodes are properly moistened, Sensation of a short light flash may occur when the switch is turned on or off abruptly, and we will ensure switching is not done abruptly to avoid this sensation. If dizziness, headaches or pain occur, adjustments can be made for proper electrode placement, and over the counter pain relievers such as acetaminophen can help. If subjects experience any changes in their mood, sleep, nausea or fatigue, we will work with their inpatient team for further assessment and management. We will ensure that all tDCS treatments are properly administered by trained staff who will remain with subject during the tDCS treatments and adjust the device

or electrode placement and settings as appropriate. Every effort will be made to ensure subjects are comfortable.

Risks of wireless EEG

Discomfort, skin irritation or allergic reaction may occur with the wireless EEG cap that contains scalp electrodes and worn over the head. Every effort will be made to ensure subjects are comfortable.

Risks from NIRSIT device

Discomfort or skin sensitivity may occur when the NIRSIT device is worn over the head. Every effort will be made to ensure the subject is comfortable.

Risks from questionnaires and neuropsychological testing

Frustration, fatigue, or discomfort with the questionnaires and testing can occur. Subjects are free to skip any question they do not feel comfortable answering. Subjects are free to take breaks and rest as needed. The depression questionnaire (MADRS) responses are screened real-time by study coordinators. Any positive response will be addressed promptly by the study clinicians, and we will work with their inpatient team for further assessment and management.

As with all research, there is a chance that confidentiality could be compromised; however, we take precautions to minimize this risk.

Depression Management Plan

The depression questionnaire (MADRS) responses are screened real-time by study coordinators. In the locked inpatient psychiatric unit, we anticipate the presence of severe depression and/or suicidal ideation which is consistent with TRD. Any positive (that is, non-zero) response on the MADRS suicidal thoughts question (#10) will be communicated by the study coordinator to the inpatient treatment team. The inpatient treatment team will be clinically assessing the patient every day, including for suicidal ideation, and further management is done as per standard clinical practice.

Non-Significant Risk Device

In our study, the tDCS device is being used as an add-on treatment in patients who are hospitalized with TRD and receiving standard clinical psychiatric and medical care in the inpatient setting. The tDCS does not replace standard care. It is non-invasive and treatments are administered by delivering low energy electric currents to the brain through electrodes placed on the scalp. Studies involving tDCS devices are generally considered Non-Significant Risk (NSR) by the FDA. [25]

The **tDCS device meets criteria for NSR** device in our study based on the following:

1. Does not present a potential for serious risk to the health, safety, or welfare of a subject.

2. Not intended as an implant.
3. Not used in supporting or sustaining human life.
4. Not used in diagnosing, curing, mitigating or treating disease.

1.4.3 Potential Benefits

tDCS is an add-on to clinical standard of care in the inpatient psychiatric setting. Subjects may experience the therapeutic benefit of improvement in depressive symptoms. Psychiatric symptoms will be assessed routinely throughout the study, in addition to routine standard of care assessment and clinical care they are receiving in the hospital setting.

Given the mild and transient nature of the adverse effects of tDCS as reported in the literature, the risks of the proposed tDCS treatment course appear to be significantly outweighed by the potential benefit for improvement in depressive symptoms.

1.5 Anticipated Duration of the Clinical Investigation

Estimated duration of the study is 1-2 weeks. Each subject is anticipated to be enrolled for up to 2 weeks. Participation will be divided into baseline assessments, tDCS treatment period (10 sessions in 5 days), and post-tDCS assessments.

2 Study Objectives

2.1 Primary Objective

2.1: The primary objective is to test the feasibility, acceptability and tolerability of a 5-day tDCS treatment protocol in individuals with TRD during the course of an acute psychiatric hospitalization, to be determined by the question “Can this study be done?”

Hypothesis 2.1: We hypothesize that the proposed 5-day tDCS treatment protocol will be feasible (enrollment of 70% of eligible patients), acceptable (80% of study participants completing 80% of the 10-session tDCS treatments) and tolerable (overall tolerability rating of "very tolerable" by self-report) to implement in the inpatient psychiatric setting.

2.2 Exploratory Objectives

2.2.1: To assess the preliminary effects of a 5-day tDCS treatment protocol on depressive and cognitive symptoms in individuals with TRD.

Hypothesis 2.2.1a: We hypothesize that patients treated with the 5-day tDCS treatment protocol will have a decrease in mean Montgomery-Asberg Depression Rating Scale (MADRS) scores post-tDCS.

Hypothesis 2.2.1b: We hypothesize that patients treated with the 5-day tDCS treatment protocol will demonstrate changes in cognitive measures (Stroop Test, Revised Hopkins Verbal Learning Test (HVLT-R), and Digital Symbol Coding Test (DSCT)) post-tDCS.

2.2.2: To explore EEG changes in peak alpha frequency (PAF) data as a potential biomarker to measure desired brain stimulation effects of tDCS in the stimulated brain region.

Hypotheses 2.2.2.a: We hypothesize that the baseline PAF will have an indirect correlation with baseline MADRS scores in depressed patients.

Hypothesis 2.2.2b: We hypothesize that there will be an increase in mean PAF scores post-tDCS.

2.2.3: To explore cerebral hemodynamic changes as measured by functional near-infrared spectroscopy (fNIRS) as a potential biomarker to measure desired brain stimulation effects of tDCS in the stimulated brain region.

Hypotheses 2.2.3a: We hypothesize that there will be an increase in cerebral hemodynamics post-tDCS.

3 Study Design

3.1 General Design

In this feasibility study we propose to conduct a 5-day tDCS treatment protocol in hospitalized adult patients with TRD, with each treatment session lasting for 30 minutes, delivered twice daily, for a total of 10 stimulations over 5 days. (Figure 1)

Figure 1. 5-day tDCS treatment protocol

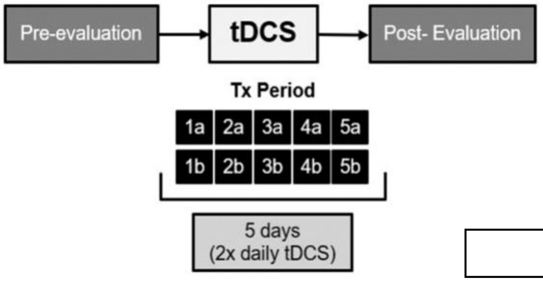

Figure 2. Study schema

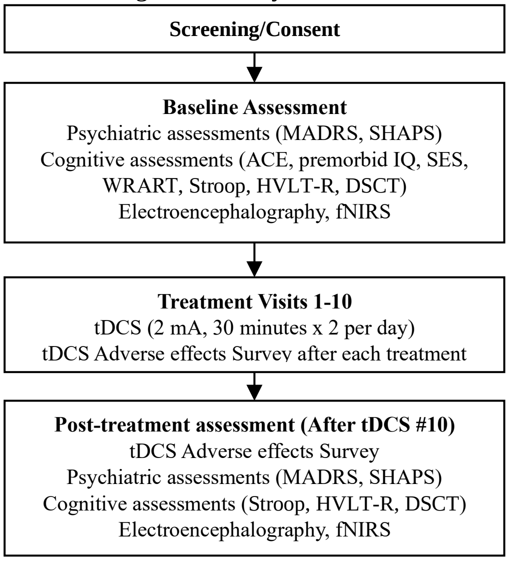

Before and after the 5-day tDCS treatment protocol, assessments will include Montgomery-Asberg Depression Rating Scale (MADRS) to measure depressive symptoms, Snaith-Hamilton Pleasure Scale (SHAPS) to measure anhedonia, Stroop Test to measure memory and executive function, Revised Hopkins Verbal Learning Test (HVLT-R) to test verbal learning and memory, and Digital Symbol Coding Test (DSCT) to measure working memory. (Figure 2) EEG for objective electrophysiologic data will also be collected before and after tDCS. A wireless EEG unit (CGX, Cognionic, Inc.) (Section 5)

will be used to collect EEG and peak alpha frequency (PAF) as potential target engagement marker for tDCS. The PAF measures the highest magnitude within the alpha range of brain oscillations, thought to reflect cognitive performance. Cerebral hemodynamics using fNIRS with the NIRSIT system (OBELAB Inc.) will be measured before and after tDCS. fNIRS indirectly measures cortical oxygenated and deoxygenated hemoglobin levels, providing insights into neural activation before and after brain stimulation. Other potential predictors of treatment response measures collected at baseline include Adverse Childhood Experiences (ACE), premorbid intelligence quotient (pIQ), Wide Range Achievement Reading Test (WRART), and Socioeconomic Status (SES).

3.2 Primary Study Endpoints

The primary goal of this study is feasibility, acceptability, and tolerability of administering a 5-day tDCS treatment protocol in acutely hospitalized TRD patients.

Feasibility

We define feasibility as 70% of eligible patients choosing to enroll in the study. Other feasibility parameters will include willingness of clinicians to refer and help recruit patients, number of eligible participants, willingness of participants to enroll, retention of patients, and completeness of data collection.

Acceptability

We define acceptability as 80% of study participants completing 80% of the 10-session tDCS treatment protocol. We will also ask participants about their satisfaction, perceptions, and experiences with the tDCS treatments. Other acceptability parameters will include rates of adherence to tDCS protocol, dropout rates and reasons for non-completion.

Tolerability

We define tolerability as an overall tolerability rating of "very tolerable." This will be a self-report based on a global question "Considering all aspects of your experience, how would you rate the overall tolerability of the tDCS intervention?" The response options are: very tolerable, somewhat tolerable, neutral, somewhat intolerable, and very intolerable. We will ask this question after the last tDCS session. Other tolerability parameters will include the tDCS adverse effects questionnaire.

3.3 Exploratory Study Endpoints

The exploratory endpoint for this study will be to observe the changes in measures of depressive symptoms based on Montgomery-Asberg Depression Rating Scale (MADRS) and anhedonia based on Snaith-Hamilton Pleasure Scale (SHAPS); cognitive measures including Stroop Test, Revised Hopkins Verbal Learning Test (HVLT-R), and Digital Symbol Coding Test (DSCT). Response is defined by a 50% or more reduction in MADRS score.

An exploratory marker of response to tDCS is EEG peak alpha frequency (PAF) which will be measured pre- and post-tDCS. Another exploratory marker of response to tDCS is cerebral hemodynamic changes pre- and post-tDCS as measured by fNIRS using the NIRSIT system (OBELAB Inc.). Other exploratory predictors of treatment response include Adverse Childhood Experiences (ACE), premorbid intelligence quotient (pIQ), Wide Range Achievement Reading Test (WRART), and Socioeconomic Status (SES). These measures will only need to be administered once at the pre-treatment assessment.

3.4 Primary Safety Endpoints

Monitoring for adverse events will be conducted during study period. Subjects will be asked to complete the tDCS Adverse Effects questionnaire after each tDCS treatment. Overall incidence of all AEs and SAEs, as well as incidence of specific AEs and SAEs, will be determined.

The tDCS adverse effects questionnaire lists 12 possible tDCS side effects and asks a participant to rate whether they experienced any on a 4-point Likert scale (ranging from 1 - absent to 4 - severe), and to what extent they attribute each side effect to tDCS (ranging from 1 - not at all to 4 - completely).[11]

4 Subject Selection, Enrollment and Withdrawal

Adult patients hospitalized in Generose will be eligible for the study.

4.1 Inclusion Criteria

- 18 years and older
- Clinical diagnosis of treatment-resistant depression (defined as depression that does not remit following two or more treatment attempts of an adequate dose and duration of a minimum duration of 4 weeks)
- Hospitalized in Generose Psychiatric Units
- Voluntary admission status
- Moderate or severe depression, defined by PHQ-9 $\geq$ 15
- Ability to provide informed consent
- Ability to adhere to protocol

4.2 Exclusion Criteria

- Bipolar disorder
- Active primary psychotic or substance use disorders (except nicotine dependence) within the past year
- Any active neurological condition (including seizure disorder, traumatic brain injury, stroke)
- Contraindications to tDCS (including pacemaker, metallic implants in the head or neck [except orthodontic hardware], skin disease causing irritation)
- Current pregnancy or positive urine pregnancy test (clinical)
- Any neuromodulation therapy (including ECT, rTMS, DBS, VNS, TES) within the last 3 months

4.3 Subject Recruitment, Enrollment and Screening

To ensure representativeness of an inpatient population, potential subjects will be recruited from both GE3W (Mood Disorders Unit where patients typically have TRD) and GE3E (Medical Psychiatric and Geriatric Psychiatric Unit where patients either have medical-psychiatric conditions or are older adults 65 years and older) inpatient units. Other than age requirement of 18 years or older and ability to provide informed consent, patients who meet inclusion criteria and are willing to participate will be enrolled regardless of sex and gender, race, or ethnic group and social determinants.

Study coordinators will review Epic for daily admissions to the inpatient units to pre-screen for eligibility. When a potential subject is identified, the study coordinators will review with the PI or Co-Investigator for appropriateness, and then ask permission from the primary inpatient treatment team consultant or providers to approach the patient. With the primary service's permission, the study coordinator will explain the study to the patient, answer questions about the study, and enroll and obtain written informed consent from the patient.

4.4 Early Withdrawal of Subjects

4.4.1 When and How to Withdraw Subjects

Subjects will be withdrawn from the study prior to completing study procedures in the following circumstances:

- Subject decision to withdraw from the study (withdrawal of consent)
- Subject inability to adhere to protocol requirements
- Severe adverse effects (SAEs) requiring medical intervention
- If the investigators determine it is in the best interest of the subject

There are no anticipated risks particular to early or abrupt discontinuation of tDCS. If subjects discontinue tDCS treatments or withdraw early and require ongoing medical treatment, appropriate referrals will be arranged. Should a subject experience any medical emergency during participation in a study visit, whether related to the study or not, the subject will be evaluated by clinical on-call inpatient psychiatric staff.

If an enrolled subject withdraws from the study prior to completion, an attempt will be made to enroll another subject in order to achieve the target enrollment.

4.4.2 Data Collection and Follow-up for Withdrawn Subjects

Data collected from subjects who withdraw from the study (whether due to subject withdrawal or withdrawal by the investigators) prior to the time of withdrawal will be included in data analyses. Subjects who discontinue tDCS treatments will be invited to continue participation in the post-tDCS assessments and this data will be collected with informed consent.

5 Study Device

5.1 Description

tDCS device

The Soterix Medical 1×1 Low Intensity Transcranial DC Stimulator Model 1300A is an investigational device manufactured by Soterix Medical, Inc. (New York, NY, USA). It is designed for use in noninvasive transcranial stimulation of the brain by delivering low-intensity electrical current to the scalp through two electrodes. (Figure 3)

The stimulator is comprised of a small control box (length: 7.91 in., width: 5.9 in., height: 2.83 in.) that contains the power source (two 9-volt alkaline batteries), displays (actual current delivered, impedance/contact quality, stimulation time remaining, low battery indicator), and controls (power, current intensity, stimulation duration, stimulation "start", and emergency abort). The device's maximum output voltage is 40 V ± 5%, and maximum output current is 2000 µA (2 mA) DC ± 1%.

Figure 3. tDCS and electrode placement

Figure 4. Electrode cables connected to tDCS stimulator

The stimulator is connected to each electrode (an anode and a cathode) by a color-coded, rubber-coated cable. (Figure 4) The conductive rubber electrode inset pads are covered by 5 × 7 cm (35 cm^2) sponges that are held against the scalp by a pair of adjustable, hypoallergenic/latex-free elastic bands (3 cm wide) or rubberized head straps. The sponge covers are soaked in normal saline (0.9% NaCl) prior to stimulation. The electrode insets, electrode cables, sponge covers, and elastic bands/head straps are supplied with the device by the manufacturer.

Wireless EEG device

EEG is the measurement, through the use of sensors and amplifiers, of scalp surface electrical potentials arising from cortical activity. The Quick-20r is a wireless, battery-operated EEG headset utilizing dry sensor technology. It allows the international 10-20 system standard channel positions/electrode placement across the scalp.

The 10-20 system derives its name from measurements of 10% and 20% of the distance between landmarks on the head, specifically, the depression above the bridge of the nose (nasion), the bump at the back of the head (inion), and the depressions in front of each ear (preauricular points). Sensors are labeled with a combination of letters and numbers indicating their respective locations, with even numbers on the right and odd numbers on the left side of the head. Midline locations are marked with a "z" for "zero." For instance, Fz sits on the frontal midline, while F3 sits left of and F4 sits right of Fz.

The headset provides an integrated approach to the wireless acquisition of recorded EEG signals. (Figure 5) Wireless technology allows the subject to move about while real-time data is collected and displayed. The headset obtains high-quality EEG with minimal scalp preparation. Patented mechanisms and replaceable dry sensors align to various head shapes and sizes, maintaining sensor positions in a standard montage. EEG channels are sampled at a time resolution of 500Hz and converted to digital data at 24 bits of resolution. The Quick-20r provides research-grade signal quality and is ideally suited for general-purpose EEG and ERP research in laboratory and field environments.

Figure 5. CGX wireless EEG

NIRSIT

The NIRSIT device is a portable functional near-infrared spectroscopy (fNIRS) device used in this study to transcutaneously measure changes in oxyhemoglobin in the prefrontal cortex using light detection. It is designed to measure variations in cerebral hemodynamics on a realtime basis by radiating a near light beam, at two wavelengths of 780nm and 850nm of laser, into the cerebral cortex. The system comes with its dedicated monitoring and analysis software. (Figure 6)

Figure 6. NIRSIT device system

During the study, the subject wears the NIRSIT, and the researcher runs the software on the tablet screen to measure changes in the subject's oxyhemoglobin. After wearing the NIRSIT, changes in oxyhemoglobin are measured using near-infrared spectroscopy during the word fluency test.

5.2 Method for Assigning Subjects to Treatment Groups

As this is an open-label, pilot trial, no randomization or assignment to different treatment groups will occur. All subjects will receive an identical course of ten 30-minute tDCS treatments at 2 mA over the course of 5 days.

5.3 Preparation and Administration of Investigational Device

tDCS Preparation

- The subject will be greeted and seated comfortably.
- Details of the subject's session will be recorded in the study file.
- Subjects will be instructed to turn off all cell phones and pagers. Eyeglasses will be removed. Subjects will be instructed to remove any metal objects above the neckline (with the exception of orthodontic hardware), particularly hairpins or large metallic earrings. Subjects will be instructed to sit quietly with their eyes open throughout the procedures.
- The left dorsolateral prefrontal cortex (L-DLPFC) position will be established in relation to external cephalic landmarks. Using a tape measure, the shortest midline distance over the scalp from the subject's nasion to the inion will be measured. The midpoint of this line will be marked on the scalp with a felt-tipped marker or grease pencil. A second line will be measured between the subject's left and right preauricular points (overlying the temporomandibular joints), passing through the midpoint of the nasion-inion line. The intersection of the nasion-inion line and the left-right preauricular line will be defined as the vertex. From the vertex, a point 5 cm lateral to the nasion-inion line (to the subject's left) and 5 cm anterior to the preauricular line will be marked with the marker/pencil. This will be the L-DLPFC reference point.
- The skin surrounding the L-DLPFC point on the subject's scalp and the right supraorbital area on the right forehead will be visually inspected. If free of visible skin injury, the skin will be cleaned with isopropyl alcohol wipes and allowed to dry.
- The stimulator's power switch will be turned to "ON".
- The electrode cables will be connected to the stimulator, with the red cable connected to the red receiver (labeled "ANODE") and the black cable connected to the black receiver (labeled "CATHODE").
- The conductive rubber electrode insets will be inspected visually for any defects and replaced if necessary. One electrode inset will be connected to the anode cable, and one electrode inset will be connected to the cathode cable.
- Two electrode sponge covers will be soaked in normal saline (0.9% NaCl), using approximately 12 mL of saline per sponge. Sponges are to be saturated but not dripping; if dripping they will be squeezed gently until no longer dripping saline.
- The electrode insets, connected to the electrode cables, will be slid into the sponges until the rubber inset is no longer protruding from the sponge.
- The electrodes (conductive rubber electrode inset enclosed by the saline-soaked sponge cover) will be placed on the scalp, with the anode (connected to the stimulator by the red cable) centered on the marked L-DLPFC reference point and the cathode (connected to the stimulator by the black cable) over the right supraorbital area. The operator will ensure that the two sponge covers are at least 5 cm apart in order to avoid forming a short circuit (electrical shunt) along the scalp.
- The electrodes will be held in place by the elastic bands or head straps. Latex-free bands will be used for patients with latex allergies.

tDCS Operation

- The operator will verify good contact of the electrodes with the skin using the stimulator's "CONTACT QUALITY" meter. If contact quality is inadequate (less than 50% of maximum contact quality), the operator will adjust electrodes, add a small amount of saline to the electrode sponge covers with a syringe, and/or clean the skin with alcohol wipes again until good contact is established.
- Current intensity will be set at 1 mA using the stimulator's "CURRENT INTENSITY" knob.
- Stimulation duration will be set at 30 minutes using the stimulator's "DURATION" knob.
- The sham control switch (used for sham-controlled trials, not applicable to this study) will be set in the "OFF" position.
- The "RELAX" sliding switch will be set to "FULL CURRENT" (i.e., the full amount set with the "CURRENT INTENSITY" knob in earlier step).
- The operator will give a "preview" of the stimulation sensation by pressing the "PRE-STIM TICKLE" button, which delivers 1 mA of current for 30 seconds prior to the main treatment stimulation. This is intended to provide reassurance for the subject and to ensure initial tolerability. This will be performed only during the first treatment visit.
- To begin the treatment stimulation, the operator will press the "START" button. This will commence a 30-second period during which the stimulator automatically and gradually escalates the current to the full intensity (2 mA), followed by full-intensity DC stimulation for the set duration (30 minutes), and concluding with a 30-second period during which the stimulator automatically decreases current intensity to zero.
- During the treatment session, the subject will be asked to remain seated, relaxed and with eyes open, and to refrain from major voluntary motor activity and conversation (unless communicating with the operator about any discomfort).
- The operator will monitor the subject and the stimulator's actual current and contact quality displays throughout the treatment session.
- If the subject experiences significant discomfort but wishes to proceed with stimulation, the operator can reduce current intensity by sliding the "RELAX" switch to a lower setting. If the subject cannot tolerate the current at full intensity, the operator will attempt to reduce current by 0.2 mA increments (as shown on the stimulator's actual current display) until tolerated.
- Should the stimulation need to be discontinued immediately for any reason, the operator will press the "ABORT" button.
- Following the conclusion of the stimulation session, the electrode assemblies and head straps will be removed from the subject, the stimulator's power will be turned off, and the electrode components and cables will be disconnected. Cables and rubber electrode insets will be cleaned with alcohol wipes between sessions.
- The operator will inspect visually the subject's skin under the electrodes to determine if there is any indication of injury.

EEG placement on the subject's head

- Grasp each handle and gently pull them apart to open the headset.
- Slide the headset over the subject's head.

- Adjust the position of the headset so the earpads fit around the subject's ears .
- Brush aside excess hair near and underneath the earpads.
- Position Fp1 and Fp2 to sit 1cm above the eyebrows.
- Reorient misaligned legs and pods.
- Grasp and rotate each pod so the sensor is perpendicular to the subject's head.
- Push the headset downward so the sensor at the crown (Cz) makes firm contact with the subject's head.
- Ensure pods lie flat on the subject's head.
- Brush aside excess hair on the forehead and any other positions where Drypad sensors are installed.
- Lift the earpad to access the ear lobe and clean with alcohol.
- Clip the A1 earclip to the left earlobe.
- Option: Attach the A2 earclip to the right earlobe.
- Option: Attach ExG passive lead wire on the A2 handle for additional biometric measurements.
- Verify headset is properly positioned on the subject's head.

EEG recording

- After properly positioning the headset, obtain direct contract on all sensors before recording
- Use LEDs to assist in making contact with the scalp on each position and check the impedance measures on each channel. Green color indicates acceptable impedance within range (< 2500 kΩ)
- Click record in the software on the dedicated laptop or iPad device after reaching acceptable impedance level on all channels.
- The software will prompt the user for a file name and filetype.
    - Enter the following for the filename: tDCS_[subject number]_[pre OR post]
    - Select .eeg as filetype. (This option is only available if the laptop is used)
- Close out the current file in the software and exit program followed by pressing power button to turn off the device

NIRSIT placement on the subject's head

- Loosen the strap before wearing to ensure enough space to wear it on the subject's head.
- If the subjects has bangs on their forehead, the bangs must be lifted with one hand. (If there is hair between the sensor and the forehead, the measurement will not be performed properly.)
- Place the sensor unit on the subject's forehead with your other hand, then use the hand that lifted the bangs to pass the strap behind the head and tighten it appropriately.
- Based on the sensor position marking on the front of the product, adjust the product so that it is in the appropriate position for the study subject's eyes, nose, and eyebrows.
- Pull the strap to both sides and attach it to the velcro to secure it.

NIRSIT Measurement

- Run NIRSIT software
    a. Select the NIRSIT EYE icon on the tablet screen. The NIRSIT software runs.
- Observer account login
    a. On the LOG IN screen, enter the email address and password of the observer to log in, and select DONE at the bottom of the screen.
    b. Select the name of NIRSIT to connect from the list that appears on the screen and then select the CONNECT button at the bottom of the screen.
- NIRSIT measurement preparation
    a. Select the registered research subject on the SELECT SUBJECT screen.
    b. Select START. Start CALIBRATION. During CALIBRATION operation, there should be as little movement as possible.
    c. When calibration is completed, the distribution of signal-to-noise ratio values of each measurement channel is displayed as a red and blue histogram, and if the initial reference value is greater than 30, the passed channels are displayed as yellow circles.
- Task Mode
    a. Select VFT on the TASK mode screen and then select START.
    b. Select Go to perform a verbal fluency test.
- During the NIRSIT measurement the respiratory pattern and photoplethysmography will be measured to regress out the physiological noise (Biopac System Inc.).

5.4 Subject Compliance Monitoring

Subjects will be monitored throughout the entirety of all tDCS treatment sessions by study staff. This will ensure that effective tDCS is delivered (e.g., good electrode contact).
As noted above, the operator can reduce current intensity if the subject experiences significant discomfort. The goal will be to ensure stimulation at the highest current intensity (up to 2 mA) that the subject finds comfortable. If the current intensity is reduced in one session, the operator will attempt to increase the current intensity back to 1 mA (or the maximum tolerated by the subject) in the following 1-2 sessions if feasible.

Adherence to the treatment will be defined as completing a minimum of 8 treatments per week of the treatment course. If a subject misses 2 treatment session in a given week, another 2 treatment visits will be offered. If a subject misses more than two treatment sessions in a week, he or she will be withdrawn from the study. Should that occur, the subject will be offered the opportunity to participate in post-treatment assessments if they consent.

5.5 Prior and Concomitant Therapy

At the screening/consent visit, the subject’s medical history will be assessed by interview of the subject or review of available medical records. This includes the subject’s prior

diagnosis of medical and neurologic conditions, prior psychiatric diagnoses and treatments, prior hospitalizations and surgical procedures, and prior medication history.

There are no exclusionary medications as it is expected that subjects will undergo medication changes while in the hospital setting. Any change to a subject's psychotropic medication regimen during the 5-day tDCS treatment may occur under the direction of the primary inpatient providers and will not constitute ineligibility.

Subjects receiving psychotherapy or counseling may continue during the study, given that this is standard clinical care in the inpatient setting. A change to the subject's therapy/counseling during the 5-day tDCS treatment may occur and will not constitute ineligibility.

5.6 Packaging and Labeling

The investigational device or its package shall bear a label with the name and place of business of the manufacturer and the following statement:

"CAUTION: Investigational Device. Federal (or United States) law limits device to investigational use."

Additional contraindications, hazards, adverse effects, interfering substances or devices, warnings, and precautions as listed in the device's operator's manual are found in Attachment. These will be printed and affixed to the investigational device's storage container.

5.7 Masking/Blinding of Study

The proposed study is an open-label feasibility trial and does not have subject blinding procedures. However, study personnel performing tDCS treatments will not perform the clinical assessments/ratings on outcome measures, and vice versa.

5.8 Receiving, Storage, Distribution and Return

5.8.1 Receipt of Investigational Devices

The investigators currently possess one Soterix Medical 1×1 Low Intensity Transcranial DC Stimulator Model 1300A device. Only one device is required for the proposed study. When additional device-related supplies are required during the course of the study (additional sponge electrode covers, replacement electrode insets or cables, etc.), supplies will be ordered from Soterix and shipped to the principal investigator in the Mayo Clinic Depression Center, Generose Building 2A, Mayo Clinic Hospital – St Mary's Campus.

Upon receipt of the study treatment supplies, an inventory will be performed, and a device accountability log completed by the person accepting the shipment. Designated study staff will count and verify that the shipment contains all of the items noted in the shipping invoice. Any discrepancies, damaged, or unusable devices in a given shipment will be documented in

the study files. The investigator will notify the supplier immediately of any discrepancies, damaged, or unusable products.

5.8.2 Storage

The Soterix Medical 1×1 Low Intensity Transcranial DC Stimulator Model 1300A requires storage and operation between temperatures of 50º and 110º F (10º and 43º C), humidities of 20% and 90%, and atmospheric pressures of 20.7 in. Hg and 31.3 in. Hg (700 hPa and 1060 hPa). These limits are well beyond the temperature, humidity, and pressure extremes experienced in indoor clinic and hospital environments.

When not in use, the stimulator will be kept in a hard, padded case (supplied with the device) for protection. The device and device-related supplies (additional sponges, saline, etc.) will be kept in a secure, locked room to which only study personnel will have access in order to prevent damage to the device as well as any unintended or unauthorized use.

5.8.3 Distribution of Study Device

All subjects will be treated with the same specific Soterix Medical 1×1 Low Intensity Transcranial DC Stimulator Model 1300A device. Each subject will receive his or her own pair of sponge electrode covers (to be used throughout all ten tDCS treatments).

A device accountability log will be kept. The serial number of the device will be recorded for each treatment. An inventory of sponge electrode covers will be recorded.

5.8.4 Return or Destruction of Study Device

At routine intervals and at the completion of the study, there will be a reconciliation of devices shipped, devices utilized, and devices remaining. This reconciliation will be logged on the device accountability form, signed, and dated. Any discrepancies noted will be documented, the investigator will be notified, and an investigation will be conducted to determine the cause of the discrepancy. Devices destroyed on site will be documented in the study files.

6 Study Procedures

Overview of the tDCS intervention:

Upon enrollment, the subject will receive tDCS treatments for 30 minutes twice daily, at least 30 minutes apart. During the tDCS treatments, the subject will remain in their room, be seated in a comfortable position, and instructed to sit quietly with their eyes open throughout the procedures. The left dorsolateral prefrontal cortex (L-DLPFC) position will be established in relation to external cephalic landmarks. The electrode cables will be connected to the tDCS stimulator, placed on the scalp, with the anode (connected to the stimulator by the red cable) centered on the marked L-DLPFC reference point and the

cathode (connected to the stimulator by the black cable) over the right supraorbital area. (Figure 4)

Subjects will receive a total of 10 tDCS treatments over 5 days. Before and after the course of 10 tDCS treatments, subjects will complete depression questionnaires, cognitive testing, and EEG. Only personnel trained in tDCS will perform the treatments. tDCS operators will be present throughout the entirety of all tDCS treatments to ensure that effective tDCS is delivered and adjust current intensity for subject comfort. Safety monitoring will be done before and after each twice daily treatments to assess for adverse effects. Personnel will be trained in using the CGX wireless EEG and NIRSIT. This study will leverage existing equipment (tDCS and EEG units) from the Mayo Clinic Depression Center and emerging infrastructure of the Minnesota Precision Neuromodulation Center (Min PeNCe) provided by a Minnesota Partnership Grant.

Procedures for each study visit are detailed below:

Visit 0: Screening Visit

The Screening Visit will determine if a patient is eligible for the study:

The Study Coordinator will review electronic medical records to complete:

- Inclusion/ exclusion
- Demographics
- Vital signs
- Medical history
- Medication history
- PHQ-9

Visit 1 - Baseline Assessment visit*

The baseline visit can take up to 2 hours and will include the following:

The **Study Coordinator** will complete:

- Enrollment and consent process
- Collect physical examination from the medical records
- Adverse Events/Serious Adverse Events forms

The **Subject** will complete:

- Montgomery-Asberg Depression Rating Scale (MADRS)
- Snaith-Hamilton Pleasure Scale (SHAPS)
- Cognitive questionnaires
    - Adverse Childhood Experiences (ACE)
    - Premorbid intelligence quotient (pIQ)
    - Wide Range Achievement Reading Test (WRART)
    - Socioeconomic Status (SES)
    - Stroop Test
    - Revised Hopkins Verbal Learning Test (HVLT-R)
    - Digital Symbol Coding Test (DSCT)

- Wireless EEG with recording
    - The wireless EEG procedure will take up to 40 minutes to complete, including 15-20 minutes of EEG recording, and up to 10 minutes before and after recording for set up and removal of EEG cap.
- fNIRS with the NIRSIT device
    - The brain imaging acquisition will take 5-10 minutes to complete, including placing the device over the head and completing a verbal fluency test for a few minutes.
- tDCS treatments (2 30-minute sessions)
    - The tDCS treatment will take up to 40 minutes to complete each time, including 30 minutes of brain stimulation, and up to 5 minutes before and after stimulation for set up and removal of device.
    - There are 2 tDCS sessions per day, with each session at least 30 minutes apart.

*tDCS treatment #1 starts during the baseline visit.

Visit 2-10 – Treatment Visits
The treatment visits can take up to 2 hours and will include the following:

The **Study Coordinator** will complete:
- Adverse Events/Serious Adverse Events forms

The **Subject** will complete:
- tDCS treatments (2 30-minute sessions)
    - The tDCS treatment will take up to 40 minutes to complete each time, including 30 minutes of brain stimulation, and up to 5 minutes before and after stimulation for set up and removal of device.
    - There are 2 tDCS sessions per day, with each session at least 30 minutes apart.

Visit 10 – Post-Treatment Visit
*The post-treatment visit can take up to 2 hours and will include the following:

The **Study Coordinator** will complete:
- Adverse Events/Serious Adverse Events forms

The **Subject** will complete:
- Wireless EEG with recording
    - The wireless EEG procedure will take up to 40 minutes to complete, including 15-20 minutes of EEG recording, and up to 10 minutes before and after recording for set up and removal of EEG cap.
- tDCS treatments (2 30-minute sessions)
    - The tDCS treatment will take up to 40 minutes to complete each time, including 30 minutes of brain stimulation, and up to 5 minutes before and after stimulation for set up and removal of device.
    - There are 2 tDCS sessions per day, with each session at least 30 minutes apart.
- fNIRS with the NIRSIT device

  - The brain imaging acquisition will take 5-10 minutes to complete, including placing the device over the head and completing a verbal fluency test for a few minutes.
- Montgomery-Asberg Depression Rating Scale (MADRS)
- Snaith-Hamilton Pleasure Scale (SHAPS)
- Cognitive questionnaires
  - Stroop Test
  - Revised Hopkins Verbal Learning Test (HVLT-R)
  - Digital Symbol Coding Test (DSCT)

*Post-treatment visit occurs on tDCS treatment #10.

Schedule of Events and Timeline

**Study Activity**	**Screening (within 1 week admission)**	**Baseline (within 72 hrs. prior to treatment 1)**	**Treatment Visits**	**Post-treatment (within 72 hrs. after treatment 10)**
	**Visit 0**	**Visit 1 (tDCS #1 is same as Visit 1)**	**Visits 1-10**	**(tDCS #10 is same as Visit 10)**
Informed Consent		X		
Medical History	X			
Medication History	X			
Pregnancy Test (from EMR)	X			
Physical Exam		X		
EEG		X		X
fNIRS (NIRSIT)		X		X
tDCS (2 mA, 30 min)			X	
PHQ-9 (from EMR)	X			
MADRS		X		X
Cognitive assessments (Stroop, HVLT-R, DSCT)		X		X
Other Assessments (ACE, premorbid IQ, SES, WRART)		X		
Device Interrogation Save to Disk				
Concurrent Medications		X		
Adverse Events			X	X
Serious Adverse Events			X	X

Timeline

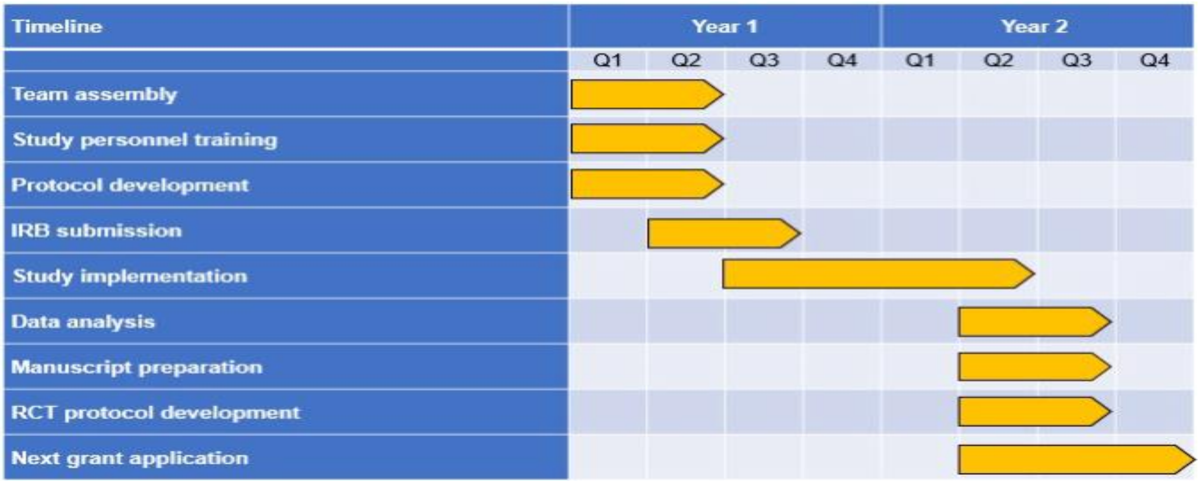

7 Statistical Plan

7.1 Sample Size Determination

Because this is a feasibility pilot study, formal power calculations are not needed. Target accrual is 10 subjects.

7.2 Statistical Methods

Descriptive Statistics

Baseline values for demographic, clinical, and outcome variables (primary and secondary) will be reported with descriptive statistics.

Handling of Missing Data

In the event where missing data cannot be prevented, we will include cases with complete data for specific analyses. In the study report, we will be transparent about the extent of missing data and the methods used to handle it to help convey the limitations of the study.

Multiplicity

Because this is a pilot study aimed at generating hypotheses, no adjustment for multiple comparisons will be made.

Primary Hypothesis:

Hypothesis 2.1: We hypothesize that the proposed 5-day tDCS treatment protocol will be feasible (enrollment of 70% of eligible patients), acceptable (80% of study participants completing 80% of the 10-session tDCS treatments) and tolerable (overall tolerability rating of "very tolerable" by self-report) to implement in the inpatient psychiatric setting.

Percentage of subjects completing the study and adhering to the protocol will be reported.

Secondary Hypothesis:

Hypothesis 2.2.1.a: We hypothesize that patients treated with the 5-day tDCS treatment protocol will have a decrease in mean MADRS scores post-tDCS.

Hypothesis 2.2.1.b: We hypothesize that patients treated with the 5-day tDCS treatment protocol will demonstrate changes in cognitive measures (Stroop Test, HVLT-R, and DSCT) post-tDCS.

T-tests or Wilcoxon signed rank tests will be used to compare pre- and post-tDCS measures on depression rating scales (MADRS, SHAPS) and cognition (Stroop, HVLT-R, DSCT). The effect sizes reported will include the proportion that improved, and both raw mean and standardized mean differences, i.e., Cohen's d.

Hypotheses 2.2.2.a: We hypothesize that the baseline PAF will have an indirect correlation with baseline MADRS scores in depressed patients.

Hypothesis 2.2.2.b: We hypothesize that there will be an increase in mean PAF scores after the 5-day tDCS treatment protocol.

Pearson or Spearman correlation will be used to estimate the correlation between MADRS scores and PAF at baseline. T-tests or Wilcoxon signed rank tests will be used to compare pre- and post-tDCS PAF scores. The effect sizes reported will include the correlation coefficient, proportion that improved, and both raw mean and standardized mean differences, i.e., Cohen's d. Treatment response will be defined as 50% or more improvement in MADRS score at post-treatment from pre-treatment. In the context of exploratory analysis, logistic regression will be used to assess associations between treatment response with each baseline characteristic.

Hypothesis 2.2.2c: We hypothesize that baseline characteristics *(ACE, pIQ, WRART, SES) will be predictive of treatment response.*

Pearson or Spearman correlation will be used to estimate the correlation between MADRS scores and PAF at baseline. T-tests or Wilcoxon signed rank tests will be used to compare pre- and post-treatment PAF scores. Treatment response will be defined as 50% or more improvement in MADRS score at post-treatment from pre-treatment. Logistic regression will be used to assess associations between treatment response with each baseline characteristic.

Interim Analysis

Because this is a feasibility pilot study, formal interim analyses are not needed.

7.3 Subject Population(s) for Analysis

For both the primary and secondary analyses, we will use the all-treated population and as a sensitivity analysis the set of subjects who completed at least 80% of 10 tDCS sessions.

8 Safety and Adverse Events

Monitoring for adverse events will be conducted during study period. Subjects will be administered tDCS Adverse Effects questionnaire after each treatment and at post-treatment assessment. Overall incidence of all AEs and SAEs, as well as incidence of specific AEs and SAEs, will be determined.

8.1 Definitions

Unanticipated Adverse Device Effect (UADE)

A UADE is any serious adverse effect on health or safety or any life-threatening problem or death caused by, or associated with, a device if that effect, problem or death was not previously identified in nature, severity, or degree of incidence in the investigational plan, or any other unanticipated serious problem associated with a device that relates to the rights, safety, or welfare of subjects.

Adverse Effect (Event)

Any untoward medical occurrence in a subject involved in clinical study of an investigational device; regardless of the causal relationship of the problem with the device or, if applicable, other study related treatment(s).

Associated with the investigational device: There is a reasonable possibility that the adverse effect may have been caused by the investigational device.

Life-threatening adverse effect: Any adverse effect that places the subject, in the view of either the investigator or the sponsor, at immediate risk of death from the effect **as it occurred**. It does not include a reaction that, had it occurred in a more severe form, might have caused death.

Serious adverse effect: An adverse effect is considered “serious” if, in the view of either the investigator or the sponsor, it results in any of the following outcomes:

- death
- a life-threatening AE
- inpatient hospitalization or prolongation of existing hospitalization
- a persistent or significant disability/incapacity
- a congenital anomaly/birth defect.

Unanticipated adverse effect: Any adverse effect, the nature, specificity, severity, or frequency of which is not consistent with the risk information in the clinical study protocol.
General Physical Examination Findings

At screening, any clinically significant abnormality should be recorded as a preexisting condition. At the end of the study, any new clinically significant findings/abnormalities that meet the definition of an adverse event must also be recorded and documented as an adverse event.

Hospitalization, Prolonged Hospitalization or Surgery

Any adverse event that results in hospitalization or prolonged hospitalization (outside of the current psychiatric hospitalization) should be documented and reported as an unanticipated adverse device effect unless specifically instructed otherwise in this protocol. Any condition responsible for surgery should be documented as an adverse event if the condition meets the criteria for an adverse event.

Neither the condition, hospitalization, prolonged hospitalization, nor surgery are reported as an adverse event in the following circumstances:

- *Hospitalization or prolonged hospitalization for diagnostic or elective surgical procedures for a preexisting condition. Surgery should* ***not*** *be reported as an outcome of an adverse event if the purpose of the surgery was elective or diagnostic and the outcome was uneventful*
- *Hospitalization or prolonged hospitalization required to allow efficacy measurement for the study*
- *Hospitalization or prolonged hospitalization for therapy of the target disease of the study, unless it is a worsening or increase in frequency of hospital admissions as judged by the clinical investigator. (This is anticipated to apply to all of our subjects who are hospitalized with TRD and can have prolonged hospitalization as indicated clinically.)*

Post-Study Adverse Event

All unresolved adverse events should be followed by the investigator until the events are resolved, the subject is lost to follow-up, or the adverse event is otherwise explained. At the last scheduled visit, the local investigator should instruct each subject to report, to the local investigator, any subsequent event(s) that the subject, or the subject's personal physician, believes might reasonably be related to participation in this study.

Preexisting Condition

A preexisting condition is one that is present at the start of the study. A preexisting condition should be recorded as an adverse event if the frequency, intensity, or the character of the condition worsens during the study period.

Unanticipated Problems Involving Risk to Subjects or Others (UPIRTSO)

Any unanticipated problem or adverse event that meets all of the following three criteria:

- Serious: Serious problems or events that results in significant harm, (which may be physical, psychological, financial, social, economic, or legal) or increased risk for the subject or others (including individuals who are not research subjects). These include: (1) death; (2) life threatening adverse experience; (3) hospitalization - inpatient, new, or prolonged; (4) disability/incapacity - persistent or significant; (5) birth defect/anomaly; (6) breach of confidentiality and (7) other problems, events, or new information (i.e. publications, DSMB reports, interim findings, product labeling change) that in the opinion of the local investigator may adversely affect the rights, safety, or welfare of the subjects or others, or substantially compromise the research data, **AND**
- Unanticipated: (i.e., unexpected) problems or events are those that are not already described as potential risks in the protocol, consent document, not listed in the Investigator's Brochure, or not part of an underlying disease. A problem or event is "unanticipated" when it was unforeseeable at the time of its occurrence. A problem or event is "unanticipated" when it occurs at an increased frequency or at an increased severity than expected, **AND**
- Related: A problem or event is "related" if it is possibly related to the research procedures.

Adverse Event Reporting Period

For this study, the study treatment period is defined as immediately following the last administration of tDCS treatment and post-tDCS assessments.

8.2 Recording of Adverse Events

At each contact with the subject, the investigator must seek information on adverse events by specific questioning and, as appropriate, by examination. Study subjects will be routinely questioned about adverse effects at study visits. Information on all adverse events should be recorded immediately in the source document, and also in the appropriate adverse event section of the case report form (CRF) or in a separate adverse event worksheet. All clearly related signs, symptoms, and abnormal diagnostic, laboratory or procedure results should recorded in the source document.

All adverse events occurring during the study period must be recorded. All observed or volunteered adverse effects (serious or non-serious) and abnormal test findings, regardless of the treatment group if applicable or suspected causal relationship to the investigational device or if applicable other study treatment or diagnostic product(s) will be recorded in the subjects' case history. For all adverse effects sufficient information will be pursued and or obtained as to permit; an adequate determination of the outcome, an assessment of the casual relationship between the adverse effect and the investigational device or, if applicable other study treatment or diagnostic product. The clinical course of each event should be followed until resolution, stabilization, or until it has been ultimately determined that the study treatment or participation is not the probable cause. Serious adverse events that are still ongoing at the end of the study period must be followed up, to determine the final outcome. Any serious adverse event that occurs after the study period and is considered to be at least

possibly related to the study treatment or study participation should be recorded and reported immediately.

Causality and Severity Assessment

The investigator will promptly review documented adverse effects and abnormal test findings to determine (1) if the abnormal test finding should be classified as an adverse effect; (2) if there is a reasonable possibility that the adverse effect was caused by the investigational device or other study treatments; and (3) if the adverse effect meets the criteria for a serious adverse effect.

If the investigator's final determination of causality is "unknown and of questionable relationship to the investigational device or other study treatments," the adverse effect will be classified as associated with the use of the investigational device or other study treatments for reporting purposes. If the investigator's final determination of causality is "unknown but not related to the investigational device or other study treatments," this determination and the rationale for the determination will be documented in the respective subject's case history.

8.3 Investigator Reporting of Unanticipated Adverse Device Effects and Unanticipated Problems

When an adverse event has been identified, the study team will take appropriate action necessary to protect the study subject and then complete the Study Adverse Event Worksheet and log. The sponsor-investigator will evaluate the event and determine the necessary follow-up and reporting required.

The investigator will promptly review documented Unanticipated Adverse Device Effects and as necessary shall report the results of such evaluation to Mayo IRB within 5 working days of initial notice of the effect. Thereafter the investigator will submit such additional reports concerning the effect as requested.

8.3.1 Investigator Reporting, Notifying Mayo IRB

The PI will report to the Mayo IRB any UPIRTSOs and Non-UPIRTSOs according to the Mayo IRB Policy and Procedures.

Deviations from the investigational plan

The PI shall notify Mayo IRB (see 21 CFR 56.108(a) (3) and (4)) of any deviation from the investigational plan to protect the life or physical well-being of a subject in an emergency. Such notice shall be given as soon as possible, but in no event later than 5 working days after the emergency occurred. Except in such an emergency, prior approval by the PI is required for changes in or deviations from a plan, and if these changes or deviations may affect the scientific soundness of the plan or the rights, safety, or welfare of human subjects and IRB notification in accordance with 21 CFR 812.35(a) also is required.

8.4 Unblinding Procedures (Breaking the Blind) (as necessary if the study is blinded)
N/A

8.5 Medical Monitoring

It is the responsibility of the investigator to oversee the safety of the study. This safety monitoring will include careful assessment and appropriate reporting of adverse events as noted above, as well as the construction and implementation of a site data and safety-monitoring plan (see Section 10 Auditing, Monitoring and Inspecting). Medical monitoring will include a regular assessment of the number and type of serious adverse events.

9 Data Handling and Record Keeping

9.1 Confidentiality

Information about study subjects will be kept confidential and managed according to the requirements of the Health Insurance Portability and Accountability Act of 1996 (HIPAA). Those regulations require a signed subject authorization informing the subject of the following:

- What protected health information (PHI) will be collected from subjects in this study
- Who will have access to that information and why
- Who will use or disclose that information
- The rights of a research subject to revoke their authorization for use of their PHI.

In the event that a subject revokes authorization to collect or use PHI, the investigator, by regulation, retains the ability to use all information collected prior to the revocation of subject authorization. For subjects that have revoked authorization to collect or use PHI, attempts should be made to obtain permission to collect at least vital status (long term survival status that the subject is alive) at the end of their scheduled study period.

9.2 Source Documents

Source data comprise all information, original records of clinical findings, observations, or other activities in a clinical trial necessary for the reconstruction and evaluation of the trial. Source data are contained in source documents. When applicable, information recorded on the CRF shall match the Source Data recorded on the Source Documents.

9.3 Case Report Forms

A Case Report Form (CRF) will be completed for each subject enrolled into the clinical study. The investigator will review, approve and sign/date each completed CRF; the investigator’s signature serving as attestation of the investigator’s responsibility for ensuring that all clinical data entered on the CRF are complete, accurate and authentic.

The study case report form (CRF) is the primary data collection instrument for the study. All data requested on the CRF must be recorded. All missing data must be explained. If a space on the CRF is left blank because the procedure was not done or the question was not asked, write "N/D". If the item is not applicable to the individual case, write "N/A". All entries should be printed legibly in black ink. If any entry error has been made, to correct such an error, draw a single straight line through the incorrect entry and enter the correct data above it. All such changes must be initialed and dated. Do not obliterate or erase errors. For clarification of illegible or uncertain entries, print the clarification above the item, then initial and date it. If the reason for the correction is not clear or needs additional explanation, neatly include the details to justify the correction.

9.4 Records Retention

The PI will maintain records and essential documents related to the conduct of the study. These will include subject case histories and regulatory documents.

The PI will retain the specified records and reports during the study and for the longer of the following;

1. As outlined in the Mayo Clinic Research Policy Manual –"Retention of and Access to Research Data Policy" http://mayocontent.mayo.edu/research-policy/MSS_669717,

   OR

2. A period of 2 years after the latter of the following two dates: The date on which the investigation is terminated or completed, or the date that the records are no longer required for purposes of supporting a premarket approval application or a notice of completion of a product development protocol.

10 Study Monitoring, Auditing, and Inspecting

10.1 Study Monitoring Plan

1. **Subject Safety**

This is a greater than a minimal risk research study. Informed consent will be obtained from all subjects before initiating study specific procedures in accordance with ethical principles, institutional policies and procedures, and federal regulations. The subject can withdraw at any time without affecting their clinical care. Adults 18 years and older are eligible to voluntarily participate in the study if they have capacity to consent to research.

Specific subject safety parameters for this study would include any adverse effects occurring while receiving tDCS. Based on prior research the main side effects could be redness of the skin or skin lesions, itching or scalp itching, scalp discomfort, tingling or burning sensations, sensation of a short light flash, dizziness, headache or pain, mood or sleep changes, nausea or fatigue.

Monitoring for adverse events will be conducted during the study period. Subjects will be administered tDCS Adverse Effects questionnaire after each treatment and at post-treatment assessment. Overall incidence of all AEs and SAEs, as well as incidence of specific AEs and SAEs, will be determined.

2. **Data Integrity**

Data monitoring is conducted to assure data is accurate and complete. Monitoring of data assures adherence to the IRB-approved protocol. The PI and study team members will be responsible for data integrity. The PI will ensure subject inclusion criteria are being met. The PI will provide oversight of entry of study data by data managers to ensure accuracy, and work with data managers in resolving any discrepancies in recorded or missing data.

3. **Subject Privacy**

An identification code will be assigned by the study staff to each subject. In order to protect the subject's identity, only the identification code will be used for any data, forms, reports, recordings, and other records.

4. **Data Confidentiality**

All paper records containing individually identifiable information and Protected Health Information (PHI) such as signed consent forms and testing results will be maintained in a secure room, and in locked file cabinets when not in use, accessible only to research personnel. There is no external data sharing of information.

5. **Product Accountability**

The tDCS equipment and supplies are available on-site. The supplies and disposables required for each treatment will be stored in a supply station separate from clinical practice stock. The study team will maintain a local device accountability log noting treatments received by each subject with the device. Reconciliation and audits will take place regularly.

6. **Study Documentation**

The PI and study team are responsible for all data collection, management and processing. All data are entered by the study coordinator into an electronic database, password-protected and only accessible to research personnel. The PI and study coordinator will review data on an ongoing basis at regularly scheduled study team meetings. The PI and study coordinator will meet with statisticians as needed during the active data collection period to conduct a formal review of the accumulating data, data quality and any data integrity issues.

7. **Study Coordination**

The PI and co-investigators are responsible for the recruitment of subjects and implementation of the study. The research interventions are performed by appropriately trained study personnel. The PI and study coordinators will meet during regularly scheduled

study team meetings to review progress and conduct of study. The PI and co-investigators will meet bi-monthly, or more frequently if needed, to review study progress and discuss any relevant issues.

11 Ethical Considerations

This study is to be conducted according to United States government regulations and Institutional research policies and procedures.

This protocol and any amendments will be submitted to a properly constituted local Institutional Review Board (IRB), in agreement with local legal prescriptions, for formal approval of the study. The decision of the IRB concerning the conduct of the study will be made in writing to the investigator before commencement of this study.

All subjects for this study will be provided a consent form describing this study and providing sufficient information for subjects to make an informed decision about their participation in this study. This consent form will be submitted with the protocol for review and approval by the IRB for the study. The formal consent of a subject, using the Approved IRB consent form, must be obtained before that subject undergoes any study procedure. The consent form must be signed and dated by the subject and the individual obtaining the informed consent.

We will ensure that each participant has the capacity to understand and voluntarily agree to participate in our study. To do this, we will rely on established clinical practices for assessing the ability to provide informed consent. First, we will actively seek the opinion of our experienced clinical team regarding each potential participant's capacity to consent. Their insights, based on familiarity with the individual's medical and cognitive status, will play a significant role in guiding our understanding and decision-making related to informed consent. Next, during the discussion with potential participants, we will provide them with detailed information about the study, its purpose, procedures, potential risks and benefits, alternatives, and their rights. We will then assess their understanding by asking open-ended questions, encourage them to ask any questions they might have, and ensure adequate time for discussion before they sign the consent form. Recognizing that a patient's clinical status can change, we will continually reassess consent throughout the study duration. If there are concerns about a patient's capacity at any point, we will revisit the consent process.

12 Study Finances

12.1 Funding Source

The study funding source is provided by the Department of Psychiatry and Psychology Small Grants Program 2023. Maria I. Lapid, M.D. (Principal Investigator/mentor) and John Li, M.D. (Co-Principal Investigator/mentee) were awarded this small grant.

12.2 Conflict of Interest

Any study team member who has a conflict of interest with this study (patent ownership, royalties, or financial gain greater than the minimum allowable by their institution, etc.) must have the conflict reviewed by a properly constituted Conflict of Interest Committee with a Committee-sanctioned conflict management plan that has been reviewed and approved by the study sponsor-investigator prior to participation in this study.

12.3 Subject Stipends or Payments

Subjects will receive up to $100 if they complete all 10 study visits. Less than 10 visits will be prorated (e.g., If they only complete 7 visits, they will receive $70). Mayo patients will be paid through the Research Participant Payment Application system (RPPA) at the end of the treatment period.

13 References

1. NIMH. *Major depression*. 2023; Available from: https://www.nimh.nih.gov/health/statistics/major-depression.
2. WHO. *Depressive disorder (depression)*. 2023; Available from: https://www.who.int/news-room/fact-sheets/detail/depression.
3. Olfson, M., et al., *Prospective Service Use and Health Care Costs of Medicaid Beneficiaries with Treatment-Resistant Depression.* J Manag Care Spec Pharm, 2018. **24**(3): p. 226-236.
4. Nemeroff, C.B., *Prevalence and management of treatment-resistant depression.* J Clin Psychiatry, 2007. **68 Suppl 8**: p. 17-25.
5. Dayan, E., et al., *Noninvasive brain stimulation: from physiology to network dynamics and back.* Nat Neurosci, 2013. **16**(7): p. 838-44.
6. Lefaucheur, J.P. and F. Wendling, *Mechanisms of action of tDCS: A brief and practical overview.* Neurophysiol Clin, 2019. **49**(4): p. 269-275.
7. Palm, U., et al., *tDCS for the treatment of depression: a comprehensive review.* Eur Arch Psychiatry Clin Neurosci, 2016. **266**(8): p. 681-694.
8. Razza, L.B., et al., *A systematic review and meta-analysis on the effects of transcranial direct current stimulation in depressive episodes.* Depress Anxiety, 2020. **37**(7): p. 594-608.
9. Klimesch, W., *EEG alpha and theta oscillations reflect cognitive and memory performance: a review and analysis.* Brain Res Brain Res Rev, 1999. **29**(2-3): p. 169-95.
10. Bruder, G.E., et al., *Brain ERPs of depressed patients to complex tones in an oddball task: relation of reduced P3 asymmetry to physical anhedonia.* Psychophysiology, 1998. **35**(1): p. 54-63.
11. Brunoni, A.R., et al., *A systematic review on reporting and assessment of adverse effects associated with transcranial direct current stimulation.* Int J Neuropsychopharmacol, 2011. **14**(8): p. 1133-45.
12. Brunoni, A.R., et al., *The sertraline vs. electrical current therapy for treating depression clinical study: results from a factorial, randomized, controlled trial.* JAMA Psychiatry, 2013. **70**(4): p. 383-91.
13. Stagg, C.J. and M.A. Nitsche, *Physiological basis of transcranial direct current stimulation.* Neuroscientist, 2011. **17**(1): p. 37-53.
14. Blumberger, D.M., et al., *A randomized double-blind sham-controlled study of transcranial direct current stimulation for treatment-resistant major depression.* Front Psychiatry, 2012. **3**: p. 74.
15. Palm, U., et al., *Transcranial direct current stimulation in treatment resistant depression: a randomized double-blind, placebo-controlled study.* Brain Stimul, 2012. **5**(3): p. 242-251.
16. Dell'Osso, B., et al., *Augmentative transcranial direct current stimulation (tDCS) in poor responder depressed patients: a follow-up study.* CNS Spectr, 2014. **19**(4): p. 347-54.
17. Brunoni, A.R., et al., *Trial of Electrical Direct-Current Therapy versus Escitalopram for Depression.* N Engl J Med, 2017. **376**(26): p. 2523-2533.

18. Aust, S., et al., *Efficacy of Augmentation of Cognitive Behavioral Therapy With Transcranial Direct Current Stimulation for Depression: A Randomized Clinical Trial.* JAMA Psychiatry, 2022. **79**(6): p. 528-537.
19. Woodham, R., et al., *Is tDCS a potential first line treatment for major depression?* Int Rev Psychiatry, 2021. **33**(3): p. 250-265.
20. Overvliet, G.M., et al., *Adverse events of repetitive transcranial magnetic stimulation in older adults with depression, a systematic review of the literature.* Int J Geriatr Psychiatry, 2021. **36**(3): p. 383-392.
21. Gaynes, B.N., et al., in *Definition of Treatment-Resistant Depression in the Medicare Population*. 2018: Rockville (MD).
22. Aparicio, L.V.M., et al., *A Systematic Review on the Acceptability and Tolerability of Transcranial Direct Current Stimulation Treatment in Neuropsychiatry Trials.* Brain Stimul, 2016. **9**(5): p. 671-681.
23. Bikson, M., et al., *Safety of Transcranial Direct Current Stimulation: Evidence Based Update 2016.* Brain Stimul, 2016. **9**(5): p. 641-661.
24. Krishnan, C., et al., *Safety of noninvasive brain stimulation in children and adolescents.* Brain Stimul, 2015. **8**(1): p. 76-87.
25. Fregni, F., et al., *Regulatory Considerations for the Clinical and Research Use of Transcranial Direct Current Stimulation (tDCS): review and recommendations from an expert panel.* Clin Res Regul Aff, 2015. **32**(1): p. 22-35.
